# Co-Designing an eHealth Service for the Co-Care of Parkinson Disease: Explorative Study of Values and Challenges

**DOI:** 10.2196/11278

**Published:** 2018-10-30

**Authors:** Åsa Revenäs, Helena Hvitfeldt Forsberg, Emma Granström, Carolina Wannheden

**Affiliations:** 1 Department of Learning, Informatics, Management and Ethics Medical Management Center Karolinska Institutet Stockholm Sweden

**Keywords:** chronic conditions, co-design, eHealth, health care, mobile phone, Parkinson disease, qualitative research, questionnaire, self-care, user involvement

## Abstract

**Background:**

The need for services to support patient self-care and patient education has been emphasized for patients with chronic conditions. People with chronic conditions may spend many hours per year in health and social care services, but the majority of time is spent in self-care. This has implications in how health care is best organized. The term co-care specifically stresses the combination of health care professionals’ and patients’ resources, supported by appropriate (digital) tools for information exchange, to achieve the best possible health outcomes for patients. Developers of electronic health (eHealth) services need to consider both parties’ specific needs for the service to be successful. Research on participants’ experiences of participating in co-design sessions is scarce.

**Objective:**

The aim of this study was to describe different stakeholders’ (people with chronic conditions, health care professionals, and facilitators) overall experiences of participating in co-design workshops aimed at designing an eHealth service for co-care for Parkinson disease, with a particular focus on the perceptions of values and challenges of co-design as well as improvement suggestions.

**Methods:**

We conducted 4 half-day co-design workshops with 7 people with Parkinson disease and 9 health care professionals. Data were collected during the workshop series using formative evaluations with participants and facilitators after each workshop, researchers’ diary notes throughout the co-design process, and a Web-based questionnaire after the final workshop. Quantitative data from the questionnaire were analyzed using descriptive statistics. Qualitative data were triangulated and analyzed inductively using qualitative content analysis.

**Results:**

Quantitative ratings showed that most participants had a positive general experience of the co-design workshops. Qualitative analysis resulted in 6 categories and 30 subcategories describing respondents’ perceptions of the values and challenges of co-design and their improvement suggestions. The categories concerned (1) desire for more stakeholder variation; (2) imbalance in the collaboration between stakeholders; (3) time investment and commitment paradox; (4) desire for both flexibility and guidance; (5) relevant workshop content, but concerns about goal achievement; and (6) hopes and doubts about future care.

**Conclusions:**

Based on the identified values and challenges, some paradoxical experiences were revealed, including (1) a desire to involve more stakeholders in co-design, while preferring to work in separate groups; (2) a desire for more preparation and discussions, while the required time investment was a concern; and (3) the experience that co-design is valuable for improving care, while there are doubts about the realization of co-care in practice. The value of co-design is not mainly about creating new services; it is about improving current practices to shape better care. The choice of methods needs to be adjusted to the stakeholder group and context, which will influence how they experience the process and outcomes of co-design.

## Introduction

The need for services to support patient self-care and patient education has been emphasized for patients with chronic conditions [1,2]. While people with chronic conditions may spend many hours in care, the majority of their time is spent in self-care. This calls for more patient-oriented and supported self-care services as well as a new type of collaborative partnership between patients and health care professionals [3,4]. The term co-care specifically stresses the need to combine health care professionals’ and patients’ resources for information exchange to achieve the best possible health outcomes for patients [5]. The use of electronic health (eHealth) tools—defined as the use of electronic means to deliver health-related information, resources, and services [6]—may be appropriate to support co-care. This paper describes the experiences of co-designing an eHealth service intended to support this type of partnership between people with chronic conditions, Parkinson disease in the specific context of this study, and health care professionals. A description of components of the intended eHealth service is beyond the scope of this paper.

Patient involvement in the improvement and development of health care services has been a key concept for many years. It has been suggested that health care services are necessarily coproduced by health care professionals and patients [4]. In parallel with coproduction, related terms, such as cocreation and co-design, have gained popularity in recent years [7]. Cocreation has been broadly defined as any act of collective creativity, while co-design signifies the span of a design process [7]. Co-design principles have been applied specifically in the development of eHealth services to support self-care in people with chronic conditions, such as in rheumatology [8], diabetes [9], oncology [10,11], and for family and carers of frail older adults [12].

Challenges and benefits of user involvement in the development of eHealth services have been described previously [13,14]. The evidence suggests a positive correlation between user involvement and system success [15]. However, research more often reports the results in terms of the service developed rather than how and to what extent the users were involved. The purpose, methods, and degree of user involvement may vary greatly between different projects. According to a structured review [16], user involvement in health care technology development is most common in the design phase of the system development lifecycle and the most common methods of user involvement include usability tests, interviews, and questionnaires, while other methods, such as design sessions or focus groups, are less common. We have identified 1 published paper that describes how experiences of co-design may differ based on team members’ roles and backgrounds [17]. More research into this area, focusing on co-design experiences of various stakeholder groups—co-design participants as well as facilitators—may add further knowledge of methodological considerations that are needed to guide co-design projects.

The aim of this study was to describe different stakeholders’ (people with chronic conditions, health care professionals, and facilitators) overall experiences of participating in co-design workshops aimed at designing an eHealth service for co-care for Parkinson disease, with a particular focus on the perceptions of values and challenges of co-design as well as improvement suggestions. The results of this study may support future research into the performance of co-design of eHealth services.

## Methods

### Study Design

We conducted 4 half-day co-design workshops in May and June 2016 to explore co-care needs among people with Parkinson disease (PwP) and health care professionals. The first 3 workshops aimed at capturing needs and generating ideas for the design of an eHealth service (see Multimedia Appendix 1). Between the 3^rd^ and 4^th^ workshops, a functional prototype was developed to visualize the ideas that had been discussed. In the 4^th^ workshop, the prototype was demonstrated as a mobile app on a smartphone and tablet (for PwP), and as a Web application (for health care professionals). The demonstration was based on a fictive scenario that captured different functionalities in a patient-provider interaction. Acceptance and usability were discussed with workshop participants.

Mainly qualitative data, but also quantitative data, were collected during and after the workshops, reflecting participants’ and facilitators’ perceptions. This study is part of an action research project that involves multiple stakeholders (academia, health care organizations, and patient organizations) in designing, implementing, and developing models of co-care for people with chronic conditions. The regional ethical committee approved the study (2015/2216-31/5).

### Participants

In this study, 7 PwP (Table 1) and 9 health care professionals (Table 2) specialized in neurology participated, together with 7 facilitators, were enrolled. All respondents reported using the Internet on a daily basis, and all but one of the PwP used a smartphone or a tablet in everyday life.

#### People with Parkinson Disease

The chairperson of the regional patient association for Parkinson disease sent an email to all registered members with a brief description of the research project. In total, 32 interested members contacted the researchers for further information. The ability to communicate in Swedish and availability for participation in all 4 workshops were the main criteria for inclusion. Variation in age, gender, and years since diagnosis were also considered. Eight PwP met the inclusion criteria and were recruited. Of these,1 dropped out prior to the first workshop. The age of the 7 participating PwP ranged from 45 to 85 years (45, 56, 68, 73, 74, 74, and 85 years).

#### Health Care Professionals

A neurologist who is a member of the research group sent an email invitation to 19 experienced health care professionals. We anticipated the recruitment of different health care professionals who are involved in the care of Parkinson disease, targeting primarily neurologists, nurses, physiotherapists, and counselors. In total, 11 health care professionals expressed interest and availability to participate and were recruited. Of these, 2 dropped out prior to workshop initiation. The age of the 9 participating health care professionals ranged from 32 to 63 years (32, 40, 45, 46, 47, 55, 58, 61, and 63 years).

Briefly, 2 professional moderators and 5 researchers with previous experience of doing co-design work in health care planned and carried out the workshops. The researchers collected data during the workshops and assisted the moderators—3 postdoctoral researchers (2 with degrees in health informatics and 1 in physiotherapy), 1 doctoral student in health care management research, and 1 research assistant (a medical doctor with a degree in public health and health informatics). The workshop facilitators collaborated with a developer who was prepared to participate in the workshops if necessary. However, collaboration between the workshop sessions was considered sufficient.

### Co-Design Workshops

#### Structure

All workshops were carried out in university facilities. Food and drinks were provided for free. The participants did not receive any other reimbursement for their participation. Each workshop was introduced by one of the researchers who informed participants about the aim and structure of the day and summarized previous achievements. The overall goals of the co-design workshops were presented as follows to the participants: (1) to identify co-care needs; (2) to agree on what an eHealth service for co-care should contain and how it should be used; and (3) to collaboratively generate ideas for an eHealth service.

The content of individual workshops was decided through an iterative process between, during, and after workshops. The facilitators used the breaks between workshop sessions to discuss progress and to decide on possible deviations from the planned schedule. At the end of each workshop, the facilitators met for a debriefing session. The researchers also sent emails to the participants to summarize achievements after each workshop. Multimedia Appendix 1 summarizes the specific aims and results of individual workshops.

**Table 1:** Characteristics and workshop attendance of people with Parkinson disease.

Identifier	Gender	Occupation	Education level	Years since diagnosis			Participation						
							Workshop 1		Workshop 2		Workshop 3		Workshop 4
P1	Female	Pension	University	>10		Present		Present		Absent		Present	
P2	Female	Pension	University	6-10		Present		Present		Present		Absent	
P3	Female	Sick leave	University	>10		Present		Present		Present		Absent	
P4	Female	Pension	University	>10		Present		Present		Absent		Present	
P5	Male	Pension	PhD	6-10		Present		Present		Present		Present	
P6	Male	Pension	PhD	1-5		Present		Present		Present		Present	
P7	Male	Sick leave	High school	<1		Present		Present		Present		Present	

**Table 2:** Characteristics and workshop attendance of health care professionals.

Identifier	Gender	Profession	Neurology experience (years)	Participation			
				Workshop 1	Workshop 2	Workshop 3	Workshop 4
H1	Female	Nurse	>10	Present	Present	Present	Present
H2	Female	Nurse	>10	Present	Present	Present	Absent
H3	Female	Nurse	>10	Present	Present	Present	Present
H4	Male	Physician	>10	Present	Present	Present	Absent
H5	Male	Physician	>10	Present	Absent	Absent	Absent
H6	Male	Physician	>10	Absent	Absent	Present	Present
H7	Female	Physician	>10	Absent	Absent	Present	Absent
H8	Female	Physiotherapist	>10	Present	Present	Present	Present
H9	Male	Physiotherapist	1-5	Present	Absent	Absent	Absent

**Figure 1:**
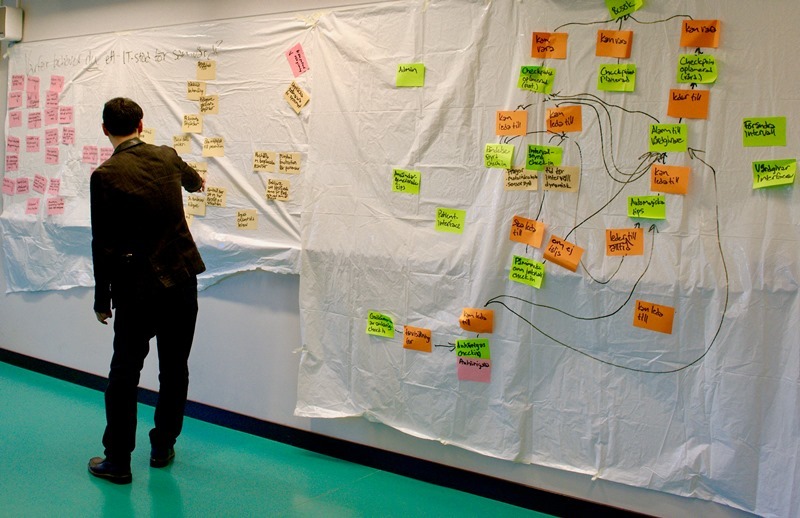
Note cards reflecting participants’ ideas in the co-design sessions.

#### Methods

The workshops contained co-design sessions and focus group discussions. The co-design sessions were used to generate, collect, and discuss ideas based on the nominal group technique [18]. First, participants were presented with a discussion question. Each participant got 3-5 note cards (depending on the question) on which they wrote down their individual reflections. The note cards were then collected and grouped according to similarities on a large canvas (Figure 1). Thereafter, a moderator led the discussion in which all individual cards were systematically discussed, rephrased, and regrouped. On 2 occasions (workshops 3 and 4), focus group discussions [19] were carried out in separate groups of PwP and health care professionals (see discussion guides in Multimedia Appendix 2).

### Data Collection

We used 3 different instruments to collect data on participants’ and facilitators’ overall experiences, perceived values, and challenges of the co-design workshops and their improvement suggestions.

#### Formative Evaluations on Note Cards

Each individual workshop was formatively evaluated using 2 questions capturing perceived values, challenges, and improvement suggestions [20]: *What worked well?* and *What could be done differently?* The participants’ feedback was collected anonymously on note cards. The facilitators provided their feedback verbally in a debriefing session after each workshop while one of the researchers took notes.

#### Researchers’ Diary

Throughout the co-design process, the researchers noted their reflections in a diary, in particular in connection with workshops and planning sessions. Unstructured diaries have been found to provide rich and in-depth data [21]. All researchers had access to the same electronic diary, and 3 of them wrote notes.

#### Summative Evaluation Using a Web-Based Questionnaire

After the final workshop, a Web-based questionnaire was distributed to all participants to collect data on their general experience of the workshops and their views on collaboration, participant contribution, and logistics. Questionnaires are a cost- and time-efficient data collection tool that offers anonymity [22]. The questionnaire in this study was designed as a structured interview guide with 7 open-ended questions, 2 ranking questions, and 2 yes or no questions (see Multimedia Appendix 3).

### Data Analysis

Descriptive statistics were used to summarize participants’ answers to 4 close-ended questions in the Web-based questionnaire, reporting percentages for the yes or no questions and the mean and range for ranking questions. Qualitative data were compiled into text documents and analyzed inductively according to principles of qualitative content analysis described elsewhere [23]. All authors read through the text. Then, 2 authors per data source coded the text separately and met to discuss and consolidate their codes. The codes (n=408) were printed out on paper slips and categorized manually. When an agreement had been reached by discussion between the researchers, the categories were transferred into mind-mapping software (FreeMind version 1.0.1) together with their constituent codes. The categorization was refined in several iterations until satisfaction was reached. Categories and subcategories were labeled to reflect the content of their constituent codes. Finally, the underlying meanings of categories were discussed, and themes were formulated. Illustrative quotes were selected to present in the results. An example of the data abstraction is presented in Multimedia Appendix 4.

## Results

### Participants’ Overall Experiences of the Co-Design Workshops

The results of quantitative analysis show that 75% (12/16) of the participants completed the questionnaire (6 of them after a reminder). All participant roles were represented—5 PwP, 3 nurses, 2 physiotherapists, and 2 physicians. The results indicated that the participants who completed the questionnaire had a positive experience regarding the co-design workshops (Table 3).

### Perceived Values, Challenges, and Suggestions for Improvements

Dataset for qualitative content analysis comprised 3 data sources as follows: (1) approximately 3000 words of diary notes from 3 researchers; (2) 165 formative workshop evaluation comments—75 of them from facilitators (based on workshops 1-4) and 92 from participants (based on workshops 1-3); and (3) 111 open-ended questionnaire responses from participants. Briefly, 6 categories and 30 subcategories that capture perceived values, challenges, and improvement suggestions were identified (Textbox 1). The 6 categories are described below, supported by illustrative quotes (translated from Swedish to English). Each quote is referenced with a unique identifier, composed of its source (wwdd: worked well; do differently feedback; d: diary; q: Web-based questionnaire) and a sequential number. In the text that follows, the descriptor *participants* refers to PwP and health care professionals; *facilitators* refers to the researchers and moderators; and *respondents* is used as an umbrella term for participants and facilitators. Individual roles, such as PwP and health care professionals, are distinguished where possible, although not for quotes from the formative feedback that was collected anonymously.

#### Desire for More Stakeholder Variation

The participants were positive about the constellation of workshop participants, representing individuals with different backgrounds and competences. They particularly valued listening to other individuals’ perspectives and opinions about care and expressed that more diversity of experiences and expertise would have been beneficial. In particular, they desired the involvement of informal caregivers and representatives from additional care professions as well as a larger PwP group with more variation in the disease status. This is reflected in the following participant quotes:

A pity there wasn’t more disease variation in the participants with Parkinson disease.

wwdd.188

Miss representatives from all care professions.

wwdd.187

**Table 3:** Results of 4 close-ended questions in the Web-based questionnaire.

Question	All participants (n=12)	PwP^a^ (n=5)	Health care professional (n=7)
“What was your overall experience of participating in the workshop series?”^b^, mean (range)	7.9 (7-9)	7.8 (7-8)	7.4 (6-9)
“In your opinion, was the workshop content in line with the aim; to develop a co-care service?”^c^ (yes), n (%)	12 (100)	5 (100)	7 (100)
“To what extent did you perceive that your voice was heard?”^d^, mean (range)	4.3 (2-5)	4 (3-5)	4.4 (2-5)
“In your opinion, was there was a balance between how much the participants with Parkinson disease and healthcare professionals voiced their thoughts?”^c^ (yes), n (%)	9 (75)	5 (100)	4 (57)

^a^PwP: people with Parkinson disease.

^b^Response scale: 1-10 (1=worst possible experience, 10=best possible experience).

^c^Response scale: yes or no.

^b^Response scale: 1-5 (1=Not at all, 5=Always).

**Categories and subcategories describing participants’ perceived values and challenges of co-design and improvement suggestions.**

Desire for more stakeholder variation (represented by participants only, P)

- Good participant constellation (P)
- Need for more diversity in expertise and experiences (P)
- Need for several representatives of individual stakeholder groups (P)

Imbalance in the collaboration among multiple stakeholders with diverse backgrounds and expectations

- Dynamic and pleasant discussion climate
- Engaged and active participants
- Differences in how much participants express their opinions
- Stakeholders managed to make their voices heard
- Communication difficulties due to differences in knowledge, roles, and expectations
- Need to balance participant activity

Time investment and commitment paradox

- Need for additional and longer workshops and more time for preparation
- Time-consuming process (P)
- Need to address patients’ health-related challenges
- Challenging to achieve long-term commitment among participants and researchers
- Need to communicate with participants before and between workshops (represented by facilitators only, F)

Desire for both flexibility and guidance from facilitators

- Need for dynamic and flexible facilitation
- Need for clearer roles and responsibilities among facilitators (F)
- Need for adequate methods for data collection during the workshops (F)
- Important to focus discussions using guidance
- Important to have good time management
- Provide clarity with appropriate tools and content
- Important to prepare the workshop setting

Relevant workshop content, but concerns about goal achievement

- General positive experiences
- Interesting and educational (P)
- Good to discuss reality and vision of care (P)
- Fun and important to co-create (P)
- Concern about workshop alignment (F)
- Inconsistent goal achievement

Hopes and doubts about future care

- Co-design creates hope for the future
- Long way to usability (P)
- Concern about health care’s readiness for co-care services

#### Imbalance in the Collaboration Among Stakeholders With Diverse Backgrounds and Expectations

On the one hand, respondents perceived that the discussion climate was dynamic and pleasant and that the participants were engaged and had a high energy level. On the other hand, they perceived that there was an imbalance in participants’ influence. Care professionals were more active in expressing their opinions, sometimes on behalf of PwP. The respondents expressed communication difficulties related to differences in knowledge and expectations about care and the health care professionals’ use of specific language that was sometimes difficult to understand for PwP. Care professionals felt that they got too much attention, but as one of them commented, “Despite imbalance, the patient group was strong and dared to contribute” (q.82). The facilitators pointed out the importance of being aware of power relationships, managing participants who dominate, and enabling silent participants (mainly PwP representatives) to speak up. One of the facilitators commented that “it felt like more participants got the chance to speak during the focus groups, which resulted in a more nuanced understanding” (d.92). This was confirmed by the participants, who favored the focus group discussions, as illustrated in the following quote by a PwP: “Maybe better with more discussions in separate groups” (q.70).

#### Time Investment and Commitment Paradox

The participants expressed a need for more time for the co-design process. They commented that more and, perhaps, longer workshops may have been beneficial. At the same time, the facilitators reflected that some of the sessions lasted too long for the participants to keep their concentration. Apart from sufficient time to collaborate in workshops, both participants and facilitators emphasized time for preparation. As a participant stated, “More time for preparation before the workshops enables us to contribute more” (wwdd.182). Meanwhile, the participants also expressed that the considerable investment of time that was required to engage in co-design was a major concern. Moreover, a care professional pointed out the need to consider health-related issues of PwP: "it would maybe have been better for patients to attend the workshops later during the day” (q.61). Facilitators noted that attendance was inconsistent for participants and for nonparticipant observers (ie, the researchers), and they reflected on how they could enhance the chances of keeping participants engaged, such as by socializing with them during breaks and maintaining contact between the workshops. Communication between workshops was appreciated by participants, as illustrated in the following quotes from health care professionals: “Useful informative mails” (q.96) and “Good to receive summaries between the workshops” (q.97).

#### Desire for Both Flexibility and Guidance From Facilitators

The respondents appreciated that the moderator was dynamic and flexible and adjusted the workshop content and structure to meet participants’ needs. As one of the health care professionals commented, “We created a structure together” (q.15). While flexibility was necessary, the facilitators recognized the need for clear roles and responsibilities and well-functioning communication channels in the team, which would also facilitate data collection for the observers. The participants appreciated when discussions were guided by concrete questions, and they emphasized the need to allow sufficient time for discussions. As one of them suggested, “Limit the scope of the tasks more in the workshops” (wwdd.179). The facilitators’ reflections indicate that they sometimes struggled with the selection of appropriate co-design methods that would support the design of an eHealth service. The participants appreciated working with note cards as they perceived that these provided a good overview of what was discussed. Furthermore, one of the facilitators reflected that “the prototype was probably key in clarifying the co-care concept” (d.103). The choice of workshop setting, characterized by sufficient space, ventilated rooms, and well-functioning technical equipment, was considered important to provide good conditions for the co-design process.

#### Relevant Workshop Content, but Concerns About Goal Achievement

The respondents shared positive experiences about the overall workshop performance, preparations, flow, logistics, structure, and teamwork. The participants perceived that the workshop content was interesting and educational, particularly when discussing the reality, expectations, and vision of care. As a participant commented, “I have a better understanding now. The workshops have helped me to reflect on the health care system in a new way” (wwdd.138). Co-design was perceived as fun and important, and one of the health care professionals noted that “It is important to develop the co-care service together to increase the chance of future use” (q.31). On the other hand, the respondents expressed concern about goal achievement, and the facilitators were concerned about succeeding in workshop alignment and ensuring progression. One of the facilitators reflected after the second workshop, “There is some concern among both the project team and participants about where we are headed” (d.42).

#### Hopes and Doubts About Future Co-Care

The respondents perceived that co-design creates hope for future care. For example, one of the participants commented on the result of the co-design workshops (ie, the eHealth prototype): “the content is promising and has potential to improve follow-up [as in continuity] of care” (wwdd.79). Nevertheless, they also realized that there is a long way to go before actual use. As one of the PwP expressed, “We took the first steps, but we have a long way to go” (q.67). The participants voiced concerns about health care’s readiness for co-care services, and one of the PwP highlighted that it is “important to engage health care” (q.106). Their concerns were that administration of a new eHealth service would take too much time and cause stress for personnel or reduce the time available for patient encounters.

## Discussion

### Principal Findings

This study explored different stakeholders’ (PwP, health care professionals, and facilitators) overall experiences of participating in co-design workshops aimed at designing an eHealth service for co-care of Parkinson disease, with a particular focus on their perceptions of the values and challenges of co-design as well as improvement suggestions. The participants had an overall positive experience of the co-design workshops. The values and challenges were identified across 6 different domains, covering, multistakeholder involvement and collaboration, time investment and commitment, flexibility and guidance from facilitators, goal achievement, and reflections on future care. A deeper analysis of the results revealed paradoxical patterns in some of the experiences, namely the following: (1) a desire to involve more stakeholders in co-design, while preferring to work in separate groups; (2) a desire for more preparation and discussions, while the required time investment was a concern; and (3) the experience that co-design is valuable for improving care, while there are doubts about the realization of co-care in practice. These paradoxes are further discussed below.

### Desire to Involve More Stakeholders While Preferring to Work in Separate Groups

The selection and recruitment of participants for the co-design workshops was a challenge because we aimed to involve diverse stakeholders, while maintaining a sensibly sized co-design group for optimal collaboration. Pearce et al describe 4 phases of user involvement: *identification*, *engagement*, *recruitment*, and *retention* [24]. We had a PwP representative and a neurologist in the project group to help us with the identification and recruitment of participants who represented central stakeholders. Informal caregivers were identified as an important stakeholder group, which was also emphasized by the participants’ feedback. The need for better support for informal caregivers has also been recognized in previous research, as they have an important role in the care of PwP [25]. However, the involvement of informal caregivers with no personal relationships with participating PwP was challenging in this study, mainly due to the timing of the workshops, which was during working hours. Furthermore, the involvement of additional stakeholders may have made it more challenging to collaborate.

While the participants’ questionnaire ratings in this study indicate that they experienced their voices being heard to a high extent and that participants’ activities were balanced, the qualitative analysis, nevertheless, indicates that health care professionals experienced that they talked too much and even spoke on behalf of PwP. This may be explained by inherent asymmetric power relationships between patients and health care professionals. The power of health care professionals has been suggested to be activated in the interaction with patients [26]. However, based on previous experiences of co-design projects with multiple stakeholders [8], the involvement of both health care professionals and patients does not necessarily inhibit patients from speaking up. During the workshop process, we became aware that there was not just a theoretical power asymmetry in the co-design group, but that there were actual patient-professional relationships among the participants. This may have hindered PwP in voicing their opinions due to the fear of possible negative consequences to their care. A good personal relationship between patients and physicians is important and has an impact on both diagnosis and treatment [27,28]. In addition, it became clear from the participants’ feedback that they appreciated and likely preferred discussions in separate groups, which may also be related to the relationship issues and power asymmetry. However, some valuable information and design ideas may only result from the interaction between the PwP and health care professionals. Thus, facilitators need to find measures to actively handle power asymmetries, similar to what has been suggested for physicians in encounters with their patients [26]. More research may be needed to expand the knowledge of potential benefits and challenges of multistakeholder collaboration in different phases of co-design. This may also require an in-depth discussion of the different stakeholder roles in co-design, including the facilitators’ roles, power relationships, and implications of partnership in co-design and co-care.

### Desire for More Preparation and Discussions While the Required Time Investment Was a Concern

A challenge experienced by the facilitators concerned the planning of co-design workshops to enable participants to engage in and commit to the entire co-design process, addressing the engagement and retaining phases of participant involvement [24]. The participants expressed concern about the co-design process being time-consuming, which is a well-known challenge of co-design [13,17,29]. During recruitment, it was particularly difficult to find health care professionals who could attend all 4 workshops, which is consistent with findings of previous research [30]. In this study, 2 of the participating health care professionals missed the first 2 workshops, which may have caused disruptions to the co-design process as they did not have the same level of understanding of the subject matter as did the other participants. However, we did not take note of such challenges. Some of the health care professionals pointed out that it was difficult for them to take time from their regular working hours. Various ways to engage participants must, therefore, be considered. In this study, we primarily used email to communicate with the participants between meetings to provide summaries from previous workshops and a plan for the next one. This was highly appreciated by the participants and enabled them to stay informed even though some of them could not attend all the workshops. Organization of additional face-to-face meetings [17] or the use of electronic means [31] has been previously discussed as a means to improve participants’ engagement. A Web-based discussion forum would enable participants to contribute their ideas or reflections throughout the co-design process. Web broadcasting in combination with a discussion forum would also enable participation from abroad. Furthermore, more silent participants could get an option to contribute their ideas electronically.

### Experience That Co-Design Is Valuable for Improving Care While Doubts About the Realization of Co-Care in Practice Are Maintained

The participants in our project raised concerns about the long way yet to go before a usable product is ready and doubts about health cares’ readiness for co-care. Previous research reports the positive impact of user involvement on system success [15,29,32-34], which may be the main driver for contributing to co-design. However, the co-design process in our project was too short for the participants to get a return on their investment of time and effort. We experienced that the main value for participants was having the opportunity to share knowledge and experiences with others and being able to contribute to the improvement of health care. Previous research has discussed that the coproduction of public services may be experienced as both empowering and exploiting by participants [35]. How to reward participant contributions and deal with intellectual property in co-design projects is not straightforward, especially if for-profit organizations are involved in the operationalization of design ideas into products and services. It is important to clarify from the beginning what results participants can expect [24] to mitigate the risk of disappointment or worse, a feeling of being exploited.

### Strengths and Limitations

The main strength of this study was the triangulation of multiple data sources collected at different timepoints to capture participants’ and facilitators’ experiences and perceptions during the co-design process. However, the data collection instruments were limited in their ability to collect in-depth data. Maybe some of the identified values and challenges were induced by the questionnaire and would not have been equally prominent if we had given the participants the opportunity to reflect more openly, such as in an interview or focus group discussion. In contrast, the diary notes from the researchers contained more in-depth reflections. Quantitative results from the questionnaire need to be interpreted with caution as the number of participants was limited and as the questionnaire was not based on previously validated items. Furthermore, variation in the degree of workshop attendance among participants may have influenced their responses.

The participation of 4 nonparticipant observers in each workshop, 1 in each corner of the room, allowed the researchers to observe group dynamics, which was important in the analysis process, even though observation notes were not included in the unit of analysis. The number of observers needs to be balanced with the risk that observation may influence participants, such as by leading them toward introspection or even questioning their own behavior [36].

### Transferability

The transferability of our findings is largely dependent on the context and how co-design is applied in the service development. There is no one-size-fits-all model [37]. The use of different approaches in different contexts naturally limits transferability. Nevertheless, we believe that our findings capture the general experiences of co-design (ie, the values and challenges that participants and researchers may experience) largely independent of the co-design aim and methods used. We have provided a rich description of our co-design process and participant characteristics to make it easier for readers to determine which of our findings may be applicable to their context. PwP in this study were on average very well educated, which may reveal an unintended selection bias. Possibly, mainly well-educated individuals felt confident to be able to participate in the project, which may also have been influenced by the location of the workshops in university facilities. We conclude that in the next phases of the project, the research team should identify other channels of participant recruitment that may lead to increased variation in participants with regard to sociodemographic factors, in general, as well as severity of disease.

### Conclusions

Our findings concerning participants’ and facilitators’ experiences of co-design are paradoxical in many ways. To generate value from co-design, the choice of methods needs to be well adjusted to the stakeholder group and to the context, which will influence how participants experience the process and outcome. Importantly, co-design is only a phase in the cocreation and coproduction of better health care, and its potential can only be realized if the generated ideas are implemented in practice. Hence, the co-design process should involve a plan for the continued engagement of stakeholders throughout the implementation process. The findings from our co-design workshops support a general need for co-care services. However, we conclude that co-design is not mainly about creating new services, but it is about improving current practices to shape better care.

## Appendix

Multimedia Appendix 1 Aims and main results of each of the 4 workshops performed during the co-design process.

Multimedia Appendix 2 Focus group discussion guides used in workshops 3 and 4.

Multimedia Appendix 3 The Web-based questionnaire sent out to participants after the final co-design workshop.

Multimedia Appendix 4 Data abstraction examples.

## Abbreviations

eHealth: electronic health
PwP: people with Parkinson disease
