# Supplementary material for: Co-Designing an eHealth Service for the Co-Care of Parkinson Disease: Explorative Study of Values and Challenges
Source: JMIR Res Protoc. 2018 Oct 30;7(10):e11278. doi: 10.2196/11278 (PMC6234336; doi:10.2196/11278)
Supplement: Multimedia Appendix 1 [file resprot_v7i10e11278_app1.pdf]

Appendix 1. Aims and main results from each of the four workshops performed during the co-design process.

Table 1 below summarizes the aims and preliminary results from each of the four workshops (WS) performed during the co-design process. Figure 1 illustrates a screenshot of the prototype that was developed based on the results from workshops 1-3.

Table 1. Summary of workshop aims and main results

WS	Aim	Main results
1	The aim was to explore co-care needs among people with Parkinson's disease (PwP) and health care professionals (important activities, functionalities).	*Identified co-care needs:*<br>• Flexible health care system<br>• Possibility to customize care<br>• Adaptation to individual needs<br>• Continuous monitoring
2	The aim was to explore the potential value of an eHealth service for co-care (why a co-care service, how to use it, three most important functionalities).	*Identified values of an eHealth service for co-care:*<br>• Improved patient safety<br>• See trends in health/illness progression<br>• Get adequate treatments<br>• Communication with health care professionals<br>*Important functions:*<br>• Functionality for sending "requests"<br>• Functionality for planned and unplanned follow-ups<br>• Overview of my health<br>• Treatment tracking: medication, physical activity<br>• Preparation for health care visits<br>• Provide tips/suggestions for better health, questions/answers
3	The aim was to decide on content and functionality to model in an eHealth prototype (based on the most frequently occurring needs and questions during care visits)	*Most common questions/issues that are discussed in a care visit:*<br>• Treatment: Medication, diet, exercise<br>• Prognosis/Progression/Disease stage<br>• Certifications for insurance/sick-leave<br>• Cause of symptoms: Is it Parkinson's disease something else causing various symptoms?<br><br>*Prioritized functions:*<br>• Provide support for medical and non-medical treatment<br>• Care planning module<br>• Functionality to prepare for visits
4	The aim was to evaluate the perceived acceptance and usability of the prototype that was designed by the design team (based on results from WS1-3) and decide on changes and next steps.	Participants were positive to the demonstrated prototype which was named *Co-Care Companion* (CCC)[a]. The prototype built on ideas from gamification to support motivation for self-care. Some of the features of CCC:<br>• Patients get assignments from their health care professionals that give points<br>• When needed, patients create requests/tickets that are sent to their health care professionals<br>• Automated tips and recommendations<br>• Continuous measurements to visualize trends<br>• Co-Care Completeness Score (0-100): visualization of the degree to which patients make use of available resources (both health care and self-care) to increase or maintain their wellbeing

[a]We aim to publish the results from the codesign process with a detailed description of co-care needs and functionality in a separate paper.

Figure 1 below illustrates a screenshot of the prototype (Co-Care Companion) that was developed based on the codesign workshops.

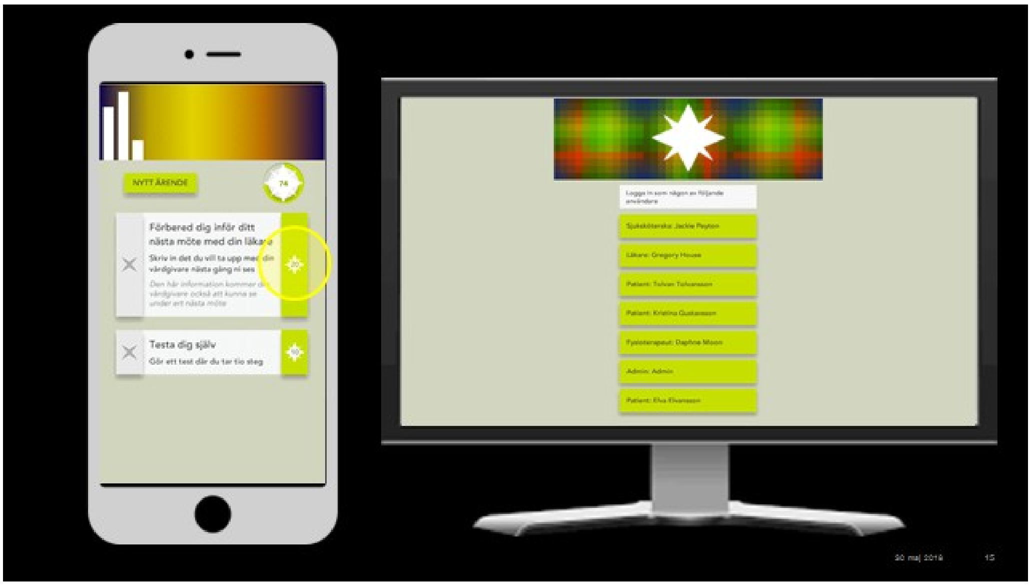

Figure 1. Screenshot of the Co-care companion. The mobile screen (left) illustrates the interface for the patients and the computer screen (right) illustrates the interface for health care professionals.
