# Supplementary material for: Co-Designing an eHealth Service for the Co-Care of Parkinson Disease: Explorative Study of Values and Challenges
Source: JMIR Res Protoc. 2018 Oct 30;7(10):e11278. doi: 10.2196/11278 (PMC6234336; doi:10.2196/11278)
Supplement: Multimedia Appendix 2 [file resprot_v7i10e11278_app2.pdf]

# Appendix 2. Focus group discussion guides used in workshops 3 and 4.

## Workshop 3, patient focus group: Experiences of a bad care visit (45 min)

*Warm-up question:*

How did you prepare for your most recent meeting with health care providers?

Round 1

- Can you tell me about a meeting with health care providers that you experienced as bad? Not because of your health, but because of the way the meeting was handled. A health care provider can be a doctor, nurse, physiotherapist or counselor, for example.

*Probing questions to use if necessary:*

- What made you think that this was bad?
- Why did you experience that as bad?
- Was there anything more you experienced as bad with that meeting?

Round 2:

- Imagine the same meeting, how could it be done differently to become a better meeting?

*Probing questions to use if necessary:*

- Would your meeting have been a better meeting then? Or would anything more have been needed?
- Just so I understand, why do you think it would have made your meeting better?
- Would that have been enough?

*Invitation questions to the group. Moderator-led discussion: everyone's reflections based on the described meetings:*

- What do you think [Name]?
- What about you [Name], would it have been enough for you if [Name]'s meeting would have been a better meeting? Do you have any other suggestions?
- Does anyone else have any suggestion how [Name]'s meeting could be better?

*If there is time / need*

Round 3:

- Is there any situation that we have mentioned where digital health technology could have improved the meeting?

Round 4:

- How do you know how you feel? Do you use any tools for monitoring your condition?

## Workshop 3, health care professional focus group: Experiences of a good care visit (45 min)

Round 1:

- Can you tell me about a meeting with a patient that you experienced as good? Not primarily because of the patient's health but in the way you handled the meeting.

*Probing questions to use if necessary:*

- What was it that made the meeting good?
- What did you do that worked?
- Why did you feel good about it?

Round 2:

- How could you transfer what you did during that patient care visit to also improve other meetings with patients?

*Invitation questions to the group. Moderator-led discussion: everyone's reflections based on the described meetings:*

- What do you think [name]?
- What do you think when you hear about [name]’s meeting? Do you see any similarities to your professional role?
- Do you think that what you did could have been supported by a digital system?

Round 3:

- It is not always the case that the patient remembers correctly or wants to tell you how they really follow their treatment.
- What do you do to know if what the patient says does not reflect reality? Can you tell us about a situation when you found out how the patient was really doing?

*Probing questions to use if necessary:*

- What did you do?
- Why do you think it got the patient to tell you the truth?
- Do you think it would work for others?
- How did you realize that you had to rephrase your question?
- Why did you think it would work?

*Invitation questions to the group. Moderator-led discussion: everyone's reflections based on the described meetings:*

- Is this something anyone else has tested as well?
- Is there anything that a digital support tool could support you with if you think about what you usually do to get the patient to tell you what their situation is really like?

## Workshop 4, patient focus group: Acceptance and perceived usability of the prototype (45 min)

*Warm up question:*

- Is there anything you would like to look at again?

*Round 1 – General impressions:*

- What are your spontaneous reactions when seeing this app? How/why?
- Is it easy or difficult to understand? How/why?
- Is there anything that is missing or excessive? How/why?

*Round 2 – Acceptance:*

- What advantages/disadvantages are there for patients? How/why?
- What barriers of use can you think of? How/why?
- Do you think that this type of app would help you to know that you have got the support you can from the health care system? How/why?

*Round 3- Usability:*

- Does an app like this seem easy to use?
- How could it be improved?
- Why would the suggested improvement make it easier to use?

*Round 4 – Conclusion:*

- Who [addressing the participants] would use this app if it were fully functional and available for use today? How/why?

## Workshop 4, health care professional focus group: Acceptance and perceived usability of the prototype (45 min)

*Warm up question:*

- Is there anything you would like to look at again?

*Round 1 – General impressions:*

- What are your spontaneous reactions when seeing this app? How/why?
- Is it easy or difficult to understand? How/why?
- Is there anything that is missing or excessive? How/why?

*Round 2 – Acceptance:*

- What advantages/disadvantages are there for patients? How/why?
- What barriers of use can you think of? How/why?
- Do you think that this type of app would support you in getting your patients to feel better? How/why?
- Do you think that this type of app would help you to know that you have got the support you can from the health care system? How/why?

*Round 3- Usability:*

- Does an app like this seem easy to use?
- How could it be improved?
- Why would the suggested improvement make it easier to use?

*Round 4 – Conclusion:*

- Who [addressing the participants] would use this app if it were fully functional and available for use today? How/why?
