# Supplementary material for: Co-Designing an eHealth Service for the Co-Care of Parkinson Disease: Explorative Study of Values and Challenges
Source: JMIR Res Protoc. 2018 Oct 30;7(10):e11278. doi: 10.2196/11278 (PMC6234336; doi:10.2196/11278)
Supplement: Multimedia Appendix 3 [file resprot_v7i10e11278_app3.pdf]

Appendix 3. The web-based questionnaire sent out to the participants after the final co-design workshop

1. What is your overall experience of participating in the workshop series? [Rating 1-10; 1 = Worst possible experience, 10 = best possible experience]
    a. Describe your experience briefly. We appreciate positive as well as negative feedback. [open-ended question]
2. In your opinion, was the workshop content in line with the aim; to develop a co-care service? [Yes/No]
3. How did you experience the discussions between patients and health care providers? Describe your experiences briefly. We appreciate positive as well as negative feedback. [open-ended question]
4. What did you appreciate with the collaboration and discussions in the workshops? [open-ended question]
5. In your opinion, what could be done better? [open-ended question]
6. To what extent did you perceive that your voice was heard? [Rating 1-5; 1= Not at all, 5=Always).
    a. Please, tell us more. [open-ended question]
7. In your opinion, was there a balance between how much the participants with Parkinson's disease and health care professionals voiced their thoughts? [Yes/No]
    a. Tell us more about your thoughts on the balance between participants' activity in the workshops. We appreciate positive as well as negative feedback. [open-ended question]
8. How did you experience the workshop logistics, i.e. information provided before and between workshops, food, workshop location, accessibility to the facilitators? We appreciate positive as well as negative feedback. [open-ended question]
9. Do you have any other feedback you want to share with us? [open-ended question]
