# Supplementary material for: Co-Designing an eHealth Service for the Co-Care of Parkinson Disease: Explorative Study of Values and Challenges
Source: JMIR Res Protoc. 2018 Oct 30;7(10):e11278. doi: 10.2196/11278 (PMC6234336; doi:10.2196/11278)
Supplement: Multimedia Appendix 4 [file resprot_v7i10e11278_app4.pdf]

Appendix 4. Data abstraction examples

Qualitative data were abstracted from three different sources: researchers' diary notes, formative evaluation notes, and open-ended web-based questionnaire responses. As the open-ended questionnaire responses contained short answers to specific questions, descriptive codes were created by adding words that described the context. Table 1 below provides an example to illustrate the data abstraction process for two sources: questionnaire and formative evaluation notes.

Table 1. Examples illustrating the data abstraction.

**Category**	**Sub-category**	**Code**	**Original text**	**Source**
Imbalance in the collaboration among multiple stakeholders with diverse backgrounds and expectations	Communication difficulties due to differences in knowledge, roles and expectations	Unbalanced discussions due to less knowledge about co-care in the patient group.	Question: Tell us more about your thoughts on the balance between participants' activity in the workshops. We appreciate positive as well as negative critique.<br><br>Answer: The patient group did not have as many [participants] as we (healthcare professionals) who had knowledge about co-care	Web-based questionnaire
Hopes and doubts about future care	Concern about healthcare's readiness for co-care services	The co-care service might take too much time for healthcare	There is a risk with co-care, that healthcare does not have time to answer patients' requests	Formative evaluation notes
